# A Novel Peptide Derived from Sea Buckthorn Leaves: Enzymatic Preparation, Dual Inhibitory Activity Against α-Glucosidase and DPP-IV, and Its Molecular Mechanism

**DOI:** 10.3390/foods15091489

**Published:** 2026-04-24

**Authors:** Xuwei Qin, Yuchong Peng, Yingqi Huang, Fang Wang, Jianfeng Guo

**Affiliations:** School of Chemistry and Chemical Engineering, Northern University of China, Taiyuan 030051, China; 15536963531@163.com (X.Q.); 13691200737@163.com (Y.P.); 18334525820@163.com (Y.H.)

**Keywords:** Sea buckthorn leaf protein, hypoglycaemic peptide, α-glucosidase, DPP-IV, molecular docking

## Abstract

Sea buckthorn leaves are a relatively underutilised component of sea buckthorn processed products; however, various studies have indicated that they possess hypoglycaemic potential. Under alkaline solubilisation and acid-precipitation conditions, the extraction yield of sea buckthorn leaf protein (SLP) reached 19.33%. Trypsin was selected as the hydrolysing enzyme to extract SLPPs-T, with half-maximal inhibitory concentrations (IC_50_) against α-glucosidase and DPP-IV of 0.1361 ± 0.017 mg/mL and 0.1286 ± 0.012 mg/mL, respectively. UV spectroscopy, Fourier transform infrared spectroscopy, circular dichroism spectroscopy and particle size analysis indicated that the secondary and microstructures of SLP underwent changes following its hydrolysis to SLPPs-T; following separation, purification, sequence identification and computer screening, two novel peptides, PM-8 and VG-11, were obtained; molecular docking, solid-phase synthesis and in vitro experiments confirmed that VG-11 exhibited superior inhibitory activity, with half-maximal inhibitory concentrations (IC_50_) against α-glucosidase and DPP-IV of 0.3885 ± 0.015 mM and 0.2611 ± 0.021 mM, respectively. In summary, this study explored the potential of sea buckthorn leaf protein as a natural hypoglycaemic product through a combination of computational modelling and experimental methods, thereby significantly enhancing the value of sea buckthorn resources.

## 1. Introduction

Sea buckthorn (*Hippophae rhamnoides* L.) is a widely distributed shrub primarily growing in Northern China and frigid regions. China possesses abundant sea buckthorn germplasm resources, accounting for approximately 90% of the global total, with cultivated sea buckthorn forests covering 55% of the nation’s total area [1]. Sea buckthorn is rich in diverse nutrients and possesses significant medicinal value, qualifying as a plant with dual medicinal and dietary applications [2]. Its fruits, seeds, and leaves all contain abundant bioactive compounds. As a component of the plant, sea buckthorn leaves are particularly rich in amino acids, vitamins, minerals, and flavonoids, exhibiting notable biological activity [3]. Currently, the development of sea buckthorn processed products primarily focuses on sea buckthorn juice, oil, and fruit powder. The leaves, as a by-product of fruit processing, remain underutilised. The development and utilisation of sea buckthorn leaves are crucial for enhancing the comprehensive utilisation of sea buckthorn waste, diversifying sea buckthorn products, and promoting the high-quality development of the sea buckthorn industry [4]. Seabuckthorn leaf protein (SLP) is the primary protein component extracted from seabuckthorn leaves. It possesses high nutritional value and bioactivity, including antioxidant, anti-inflammatory and antibacterial properties, and also exhibits beneficial immunomodulatory effects [5]. It holds significant potential for application in the food, health supplement and cosmetics sectors.

Diabetes mellitus is a chronic metabolic disorder characterised by hyperglycaemia, currently ranking as the third most prevalent chronic non-communicable disease after cancer and cardiovascular disease. Type 2 diabetes mellitus (T2DM) constitutes the most common form, accounting for over 90% of cases [6]. Controlling blood glucose levels and maintaining glycaemic homeostasis are crucial for preventing and managing diabetes and its complications. Inhibiting the activity of α-glucosidase and DPP-IV represents an effective approach to improving blood glucose control. Alpha-glucosidase inhibitors primarily act on the brush border of the small intestine. By inhibiting alpha-glucosidase activity, they slow the rate at which carbohydrates are hydrolysed to produce glucose, thereby delaying glucose absorption and reducing the postprandial rise in blood glucose levels [7]. Dipeptidyl peptidase-IV (DPP-IV) inhibitors (such as sitagliptin and alogliptin) prolong the half-life of endogenous GLP-1 by inhibiting DPP-IV enzyme activity, thereby enhancing insulin secretion and glucose degradation. These agents demonstrate unique potential, particularly in improving immune function, promoting health, and developing novel eco-friendly materials [8,9]. However, these chemical drugs may cause gastrointestinal adverse reactions (such as bloating and increased flatulence) and are not suitable for individuals with hepatic or renal impairment, pregnant women, lactating mothers, or children [10,11]. Consequently, peptides extracted from natural plant proteins through enzymatic hydrolysis are attracting increasing attention as natural inhibitors of enzymes associated with hyperglycaemia. To date, researchers have extracted α-glucosidase or DPP-IV inhibitory peptides from natural plant proteins such as soybean protein, longan seed protein and camellia seed cake protein [12,13,14]; however, the extraction of such peptides from sea buckthorn protein has never been reported.

This study evaluated the hypoglycaemic potential of sea buckthorn leaf peptides (SLPPs) obtained by proteolytic digestion of sea buckthorn leaf protein (SLP) using various proteases, particularly their inhibitory capacity against α-glucosidase and DPP-IV. Protease fractions were purified and a peptide segment exhibiting potent dual inhibition of α-glucosidase and DPP-IV was identified through peptide sequencing, peptidomics, and molecular docking. Its mechanism of action was investigated, providing a foundation for the development and application of natural hypoglycaemic peptides.

## 2. Materials and Methods

### 2.1. Materials

Sea buckthorn leaves are sourced from the Altay region of Xinjiang (Sunstone Biopharmaceuticals, Changsha, China); NaOH, HCl and anhydrous Na_2_CO_3_ were sourced from Tianjin Beichen Fangzheng Reagent Factory (Tianjin, China); alkaline protease, papain, trypsin, α-glucosidase and nitrophenyl-α-D-glucopyranoside were all sourced from Shanghai Yuanye Biotechnology Co., Ltd. (Shanghai, China); Dextran gel G-50 (separation range 1000–30,000) was sourced from Beijing Solabio Technology Co., Ltd. (Beijing, China); flavour protease was purchased from Novozymes (Copenhagen, Denmark); acarbose was purchased from Beijing Bailingwei Technology Co., Ltd. (Beijing, China); and the DPP-IV inhibitor screening assay kit was purchased from Cayman Chemical (Ann Arbor, MI, USA).

### 2.2. Extraction of Sea Buckthorn Leaf Protein

Six hundred millilitres of deionised water were added, and the mixture was stirred until homogeneous, followed by ultrasonic treatment (temperature 25 °C, power 240 W, frequency 95 40 kHz) for 20 min. The pH was adjusted to 12 using 1 mol·L^−1^ NaOH, and the mixture was stirred under alkaline conditions at 60 °C for 1 h. Following alkaline solubilisation, the sample was centrifuged at 4000× *g* r·min^−1^ (using a BIOBASE TG-18W (BIOBASE Company, Jinan, China) centrifuge with a 6 × 100 mL rotor and a maximum centrifugal 98 force of 16,390× *g*) for 20 min, and the supernatant was collected. Subsequently, the pH was adjusted to 3.5 using 1 mol·L^−1^ HCl, and the suspension was allowed to stand for 40 min, and centrifuged again at 4000× *g* 100 r·min^−1^ for 20 min to collect the precipitate. The precipitate was washed with water and centrifuged to recover the pellet, which was resuspended in water, adjusted to pH 7 using 1 mol·L^−1^ NaOH, and then centrifuged again for 20× *g* minutes to collect the supernatant. Finally, the supernatant was vacuum-free-dried to produce a freeze-dried powder of soluble sea buckthorn leaf protein (SLP).

### 2.3. Preparation of Sea Buckthorn Leaf Peptides

Sea buckthorn leaf peptides were prepared using an enzymatic hydrolysis method, based on the method described by Subhiksha Chandrasekaran et al. [15] with slight modifications. An amount of 0.20 g of freeze-dried sea buckthorn leaf protein powder was weighed out and dissolved in 10 mL of deionised water (material-to-solution ratio 1:50), resulting in four protein solutions of equal concentration. With the exception of the blank group, approximately 4000 U of enzyme was added to each group, and hydrolysis was carried out at the respective optimal temperatures.

Group 1: An amount of 5 mg of papain was added and hydrolysed at 45 °C; the resulting product was named SLPPs-P.

Group 2: An amount of 4 mg of flavour protease was added and hydrolysed at 50 °C; the product was named SLPPs-F.

Group 3: pH was adjusted to 8.0, 20 mg of alkaline protease was added, and hydrolysis was carried out at 50 °C; the resulting product was named SLPPs-A.

Group 4: pH was adjusted to 7.0, 16 mg of trypsin was added, and hydrolysis was carried out at 37 °C; the resulting product was named SLPPs-T.

During the enzymatic hydrolysis process for each group, 200 μL of the hydrolysate was sampled every hour, heat-inactivated in a 90 °C water bath for 5 min, diluted with water to 4 mL, and adjusted to a final concentration of 1 mg·mL⁻¹. The inhibitory activity of the four hydrolysates was measured at 0, 1, 2, 3, 4 and 5 h.

### 2.4. Hydrolysate Determination

The degree of protein hydrolysis was determined using the OPA method [16]. A serine standard curve was plotted using different volumes of serine standard solution. Method for determining the degree of hydrolysis: 1 mL of the enzymatic hydrolysate was taken and diluted with deionised water to a final volume of 100 mL (to ensure that the subsequent measured values fell within the range of the standard curve); 400 μL of this was pipetted into a cuvette containing 3 mL of OPA solution; after standing at room temperature for 2 min, the absorbance of the mixture was measured using a UV spectrophotometer and the procedure was repeated five times to obtain the mean value; Cserine−NH_2_ was calculated from the 133 standard curve, and the degree of hydrolysis was determine using Equations (1)–(3):

(1) $$W_{\mathrm{serine}-{\mathrm{NH}}_{2}}=C_{\mathrm{Serine}-{\mathrm{NH}}_{2}}\times \frac{V\cdot N}{X\cdot P\%}$$

(2) $$h=\frac{W_{\mathrm{serine}-{\mathrm{NH}}_{2}}-\beta }{\alpha }$$

(3) $$\mathrm{DH}=\frac{h}{h_{\mathrm{tot}}}\times 100\%$$

In the equation, $W_{\mathrm{serine}-{\mathrm{NH}}_{2}}$ (mmol·g^−1^): the amount of serine-NH_2_ per gram of protein, calculated from the serine standard curve; X (g): the mass of the analysed sample; P (%): the mass fraction of protein in the analysed sample; V (L): the volume of the hydrolysate of the analysed sample; N: the dilution factor of the hydrolysate; h (mmol·g^−1^): the number of peptide bonds cleaved per gram of protein during hydrolysis; h_tot_ (mmol·g^−1^): the total number of peptide bonds per gram of protein (SLP is 6.088 mmol/g); α and β: represented by the constants 1.00 and 0.40, respectively.

### 2.5. Determination of Inhibitory Activity Against Hyperglycaemic Enzymes

The in vitro α-glucosidase inhibitory activity assay was adapted from the method described by Mariane Daou et al. [17] with minor modifications: the reaction volume totalled 220 μL, comprising 40 μL of sample containing approximately 40 μg of enzymatically digested peptide. The positive control employed acarbose, an antidiabetic agent, as the inhibitor. A 40 μL acarbose solution, containing approximately 2 μg acarbose, was used. A 40 μL enzyme solution (1 U·mL^−1^) was mixed with 40 μL of different sample solutions/acarbose in a 96-well plate and incubated at 37 °C in an intelligent biochemical incubator for 10 min. An amount of 40 μL of 4-nitrophenyl-α-D-glucopyranoside (PNPG) was added as the reaction substrate. After incubation at 37 °C for 10 min, 100 μL of 1 mol·L^−1^ Na_2_CO_3_ solution was added to terminate the reaction. The absorbance was measured at 405 nm using an enzyme-linked immunosorbent assay reader. Deionised water was used in place of the extraction solution as the blank control. The concentration on the x-axis and the inhibition rate on the y-axis were plotted. The inhibition rate is calculated as follows. All experiments were repeated three times; results are expressed as the mean ± standard deviation. The inhibition rate was calculated using Formula (4):

(4) $$\mathrm{Inhibition}\;\mathrm{rate}\left(\%\right)=\left(1-\frac{A_{\mathrm{Sample}}-A_{\mathrm{blank}}}{A_{\mathrm{control}}}\right)\times 100\%$$

In the equation: A_sample_ denotes the absorbance after adding inhibitor and enzyme reaction; A_blank_ denotes the absorbance after adding inhibitor only, without enzyme reaction; A_control_ denotes the absorbance after adding enzyme only, without sample.

The in vitro DPP-IV inhibitory activity assay was adapted from the method described by Lingyan Dai et al. [18] with minor modifications. Three experimental groups were established: the experimental group, the control group, and the blank group. In a 96-well microplate, 30 μL buffer solution, 10 μL DPP-IV inhibitor, 10 μL sample solution, and finally 50 μL substrate (containing 100 μmol·L-1 H-Gly-Pro) were sequentially added to initiate the reaction. The plate was incubated at 37 °C for 30 min. Fluorescence intensity was measured at excitation wavelength 360 nm and emission wavelength 460 nm using a microplate reader. The control and blank groups used deionised water in place of the sample solution, while the blank group substituted the DPP-IV solution with a buffer solution. The DPP-IV inhibition rate was calculated as per Formula (5):

(5) $$\mathrm{Inhibition}\;\mathrm{rate}\left(\%\right)=\left(1-\frac{A_{\mathrm{Sample}}-A_{\mathrm{blank}}}{A_{\mathrm{control}}}\right)\times 100\%$$

In the formula, F_control _denotes the fluorescence intensity of the control group; F_blank _denotes the blank control intensity; and F_sample _denotes the fluorescence intensity of the experimental group.

### 2.6. Analysis of Extracted Protein and Peptide Properties

#### 2.6.1. Ultraviolet–Visible Spectroscopy

Liquid and dry samples of SSP and SLPPs-T were dissolved in ultrapure water to prepare 1 mg/mL solutions. UV spectra were obtained by scanning from 190 to 400 nm using a Readmax 1900 full-wavelength microplate reader (Shanpu Biotechnology Co., Ltd., Shanghai, China).

#### 2.6.2. Fourier Transform Infrared Spectroscopy (FTIR)

The secondary structure of SLP and SLPPs-T was analysed using a Fourier transform infrared spectrometer (Thermo Nicolet IS 5, Thermo Fisher Scientific company, Newark, DE, USA). A 0.2 g sample was mixed with 200 mg of potassium bromide and pressed into a transparent pellet. The scan range was 400–4000 cm^−1^, with spectral analysis performed using Origin 2024 for peak labelling.

#### 2.6.3. Circular Dichroism (CD) Spectrometer

A 0.1 mg·mL^−1^ solution of SLP and SLPPs-T was analysed using a J-1500 CD circular dichroism (CD) spectrometer (JASCO, Tokyo, Japan). The scanning wavelength range was 190–400 nm, with a scanning speed of 200 nm·min^−1^, data acquisition interval of 0.5 nm, bandwidth of 1 nm, and temperature set at 20 °C.

#### 2.6.4. Particle Size

Zeta potential measurements were conducted by diluting the sample 100-fold in 2 mg·mL^−1^ PBS solution (pH 7.4). Samples were injected into the instrument at room temperature for analysis.

### 2.7. Separation, Purification, and Molecular Weight Range of Enzymatically Cleaved Peptides

Enzymatically cleaved peptides were separated and purified using dextran gel chromatography [19]. Gel chromatography conditions: deionised water pH = 7.0, gel column dimensions 30 × 2 cm, enzyme sample volume 2.5 mL. An automated collection system gathered 0.6 mL eluate per tube, with 50 tubes collected in total. Eluates were tested for inhibitory activity; active peaks were pooled according to inhibition results to yield a purified enzyme solution.

The peptide molecular weight range was determined using the dextran gel method [20]. Within a defined range, protein elution volume exhibits good linear correlation with the logarithm of molecular weight (logMW). Using a peptidase inhibitor (6511.51 Da) and bovine trypsin (Mr = 23,800 Da) as standards, these were separately separated on a dextran gel column. Lysozyme (14,300 Da) and bovine trypsin (Mr = 23,800 Da) were separated on a dextran gel column. The elution volumes for proteins of different molecular weights were determined by measuring the optical density (OD) at 280 nm. A molecular weight–elution volume regression curve was plotted with elution volume on the x-axis and lgMr on the y-axis.

### 2.8. Determination of Half-Maximal Inhibitory Concentration (IC_50_)

The method of Wu et al. [21] was referenced with minor modifications. A specific weight of sample was weighed and prepared into solutions of varying concentrations. Inhibitory activity was measured by plotting concentration on the x-axis and inhibitory activity on the y-axis. All experiments were repeated three times, with results expressed as mean ± standard deviation. The IC_50_ value was calculated using Prism 10.6 software for curve fitting.

### 2.9. Sequence Identification of Proteins and Peptides

Sequences of sea buckthorn leaf peptides were identified using liquid chromatography-tandem mass spectrometry (LC/MS-MS). Samples underwent desalting via a C18 column. The system comprised a Q Exactive mass spectrometer (Thermo Fisher Scientific, Newark, DE, USA) coupled with tandem liquid chromatography, employing a Column Technology Inc. RP-C18 column (0.15 mm × 150 mm) (Column Technology, Lombard, IL, USA). Mobile phase A was 0.1% formic acid aqueous solution, and mobile phase B was 0.1% formic acid acetonitrile aqueous solution (acetonitrile 84%). Samples were separated over a 60 min gradient: 0–50 min: 4–50% B; 50–54 min: 50–100% B; 54–60 min: 100% B.

Enzymatically cleaved products were separated by capillary high-performance liquid chromatography and analysed by mass spectrometry using a Q Exactive mass spectrometer (Thermo Fisher Scientific, Newark, DE, USA). Analysis duration: 60 min, detection mode: positive ions. Mass-to-charge ratios of peptides and peptide fragments were acquired as follows: 10 fragment spectra (MS2 scan) collected after each full scan. Raw mass spectrometry files were analysed using MaxQuant 1.5.5.1 software against relevant databases, yielding final protein identification and quantitative results.

### 2.10. Virtual Screening

Peptide bioactivity was predicted using PeptideRanker (http://distilldeep.ucd.ie/PeptideRanker/ accessed on 5 January 2026) [22]; the higher the score, the greater the potential. A higher score indicates greater potential. StackDPPIV is a novel stack-based ensemble learning predictor designed to identify DPP-IV inhibitory peptides [23] (which achieves higher predictive accuracy than existing methods). Toxin Pred database (http://crdd.osdd.net/raghava/toxinpred/ accessed on 5 January 2026) predicted peptide toxicity, while AllerTOP V.2.0 database (https://www.ddg-pharmfac.net/AllerTOP/index.html/ accessed on 6 January 2026) assessed allergenic potential [24]. Finally, the BIOPEP-UWM database (https://biochemia.uwm.edu.pl/biopep/start_biopep.php/ accessed on 6 January 2026) was employed to screen for unreported peptides. The filtered peptides were compared against reported bioactive peptides in the database to identify novel peptides for subsequent research [25].

### 2.11. Molecular Docking

Molecular docking was performed using AutoDock Tools 1.5.7 to analyse protein–peptide interactions. The 3D structures of α-glucosidase (PDB ID: 3AJ7) and DPP-IV (PDB ID: 5J3J) were downloaded from the Protein Data Bank (PDB) of the Research Consortium on Structural Biology (RCSB). Using PyMOL 3.1.3 software, the original ligand and all water molecules were removed, and hydrogen atoms were added. The pre-treated protein was defined as the target receptor. The peptide structure was constructed using Chem3D 14.0.0.17 software and subjected to energy minimisation to optimise its structural stability. The processed peptide was categorised as the ligand for subsequent molecular docking experiments. Docking parameters were set as follows: α-glucosidase (PDB ID: 3AJ7): centre_x = 21.346, centre_y = −0.264, centre_z = 8.692; docking box dimensions: 40 × 40 × 40 Å (X, Y, Z axes);

DPP-IV (PDB ID: 5J3J): centre_x = 28.879, centre_y = −2.763, centre_z = 72.412; dimensions in the X, Y, and Z directions were 40 × 40 × 40. Following completion of the docking experiments, a total of 10 model sets were obtained. To identify the most stable protein–peptide complex conformation, the model exhibiting the lowest docking energy was selected, as lower docking energy typically indicates a more stable binding state. Finally, PyMOL software was utilised to visually inspect the docking results and generate diagrams.

### 2.12. Peptide Synthesis and In Vitro Activity Validation

Peptides were synthesised using the Fmoc solid-phase method (Qingdao Chuangfeng Zhishi Testing Technology Co., Ltd., Qingdao, Shandong, China), with in vitro activity assays conducted per Section 2.5.

### 2.13. Statistical Analysis

All experiments were conducted in triplicate, with results expressed as mean ± standard deviation. IC_50_ values were calculated using GraphPad Prism 10.1.2, whilst statistical analysis was performed with IBM SPSS Statistics version 27.

## 3. Results and Discussion

### 3.1. Extraction of Sea Buckthorn Leaf Proteins and Peptides

**Figure 1:**
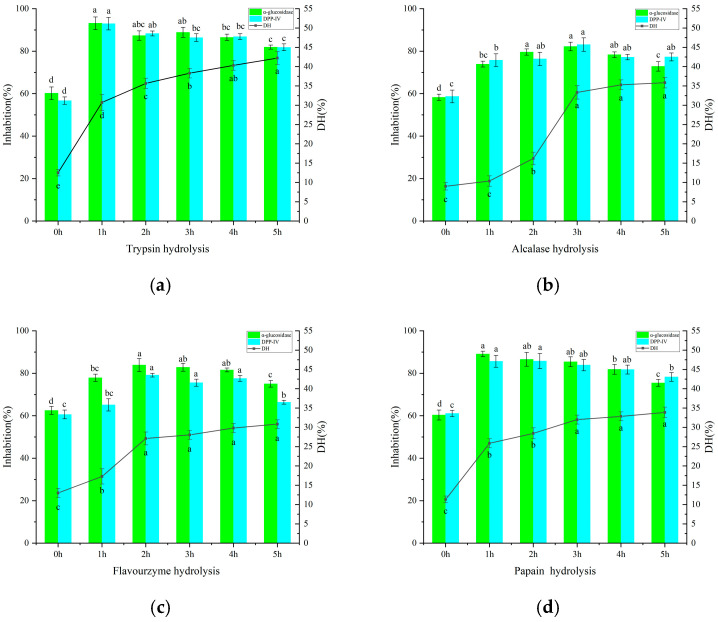
Effect of enzymatic digestion time on the hypoglycaemic activity and degree of hydrolysis of sea buckthorn leaf protein. (**a**) Trypsin digestion; (**b**) alkaline protease digestion; (**c**) flavoproteinase digestion; (**d**) papain digestion.

Proteins were extracted from sea buckthorn leaves using the alkali-solubilisation and acid-precipitation method; the yield of crude protein from 15 g of leaf powder was 4.24 g, representing 28.27% of the leaf powder’s mass. In this study, four enzymes—papain, trypsin, chymopapain and alkaline protease—were used to hydrolyse SLP over varying time periods. The hydrolytic capacity (DH) of the different enzymes towards SLP was determined, as well as the inhibitory activity of the resulting hydrolysed peptides against α-glucosidase and DPP-IV (Figure 1). The results indicate that SLP possesses a certain degree of inhibitory activity on its own; the SLPPs produced after hydrolysis by the four enzymes exhibited varying degrees of enhanced inhibitory activity against α-glucosidase and DPP-IV, suggesting that under the action of hydrolytic enzymes, SLP generates more peptide fragments with hypoglycaemic potential. As shown in Figure 1, using α-glucosidase inhibitory activity as an indicator, SLPPs-T exhibited the highest inhibitory activity against α-glucosidase, reaching 93.14% (1 h hydrolysis), followed by SLPPs-P, which exhibited an inhibitory capacity of 89.77% (1 h hydrolysis). The inhibitory capacities of SLPPs-A and SLPPs-F were weaker than those of the former two, at 82.18% (3 h hydrolysis) and 83.89% (2 h hydrolysis), respectively. When DPP-IV inhibitory activity was used as the indicator, SLPPs-T exhibited the highest DPP-IV inhibitory activity, reaching 92.93% (1 h hydrolysis), followed by SLPPs-P, which exhibited an inhibitory capacity of 85.62% (1 h hydrolysis). The inhibitory capacities of SLPPs-A and SLPPs-F were weaker than those of the former two, at 83.09% (3 h hydrolysis) and 79.12% (2 h hydrolysis), respectively. In addition to differences in their inhibitory effects on hyperglycaemic enzymes, the four proteases also exhibited varying degrees of protein hydrolysis during the hydrolysis process. Trypsin and papain showed similar hydrolysis trends: a marked increase in protein hydrolysis degree between 0 and 1 h, a relatively gentle rise between 1 and 5 h, and a slight downward trend after 1 h. For flavour protease, the degree of hydrolysis increased more markedly between 1 and 2 h, with a relatively gentle rise between 2 and 5 h; for alkaline protease, the rate of increase in the degree of hydrolysis was relatively rapid between 2 and 3 h. Considering both the degree of hydrolysis and the inhibitory effect on hyperglycaemic enzymes, the period of rapid increase in the degree of hydrolysis produces a series of peptides with different amino acid sequences and spatial structures, rapidly enhancing the inhibitory effect on hyperglycaemic enzymes; however, as the enzymatic hydrolysis reaction continues, the inhibitory effect weakens somewhat. Excessive hydrolysis may lead to the degradation or structural changes in certain inhibitory peptide segments, thereby reducing their inhibitory activity. Consequently, sea buckthorn leaf protein possesses potential hypoglycaemic properties, and enzymatic hydrolysis can release a greater number of active peptides with inhibitory effects.

In summary, SLP was extracted using the alkali-solubilisation and acid-precipitation method, and the inhibitory activities of four enzymes were compared. The peptide mixture SLPP-T, obtained by trypsin hydrolysis of SLP, demonstrated the best inhibitory effects against α-glucosidase and DPP-IV. The hydrolysis conditions were as follows: SLP was dissolved in water (solid-to-liquid ratio 1:50), with 80 mg of trypsin added per gram of protein, and hydrolysis was carried out at 37 °C to obtain SLPP-T (Different lowercase letters represent the significant analysis results of DH and inhibition by IBM SPSS Statistics 27).

### 3.2. Structural Characteristics of Sea Buckthorn Leaf Protein and Peptides

Based on the inhibitory activity results of the enzymatic hydrolysis products, sea buckthorn leaf protein (SLP) and the peptides (SLPPs-T) obtained from trypsin hydrolysis of SLP were selected for structural characterisation studies.

#### 3.2.1. Ultraviolet–Visible Spectra

**Figure 2:**
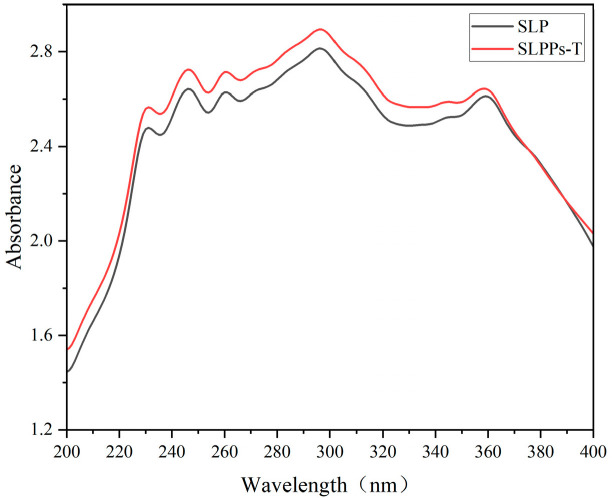
UV-Vis spectra of SLP and SLPPs-T.

Conjugated double bonds present in the chemical structure of proteins render the 280–295 nm range, the preferred region for characterising structural changes and denaturation in proteins. The near-ultraviolet region (240–295 nm) corresponds to the absorption band of side-chain residues (e.g., tyrosine, tryptophan, phenylalanine, and cysteine), with tryptophan residues exhibiting absorption due to π → π* transitions [26]. As shown in Figure 2, the absorption peaks of SLP and SLPPs-T peak at 296 nm, demonstrating that both possess distinct protein characteristics. The UV absorption spectrum of SLPPs-T is higher than that of SLP across the characteristic absorption wavelength range; this is likely due to SLP exposing more aromatic amino acids following hydrolysis.

#### 3.2.2. Secondary Structure Analysis

**Figure 3:**
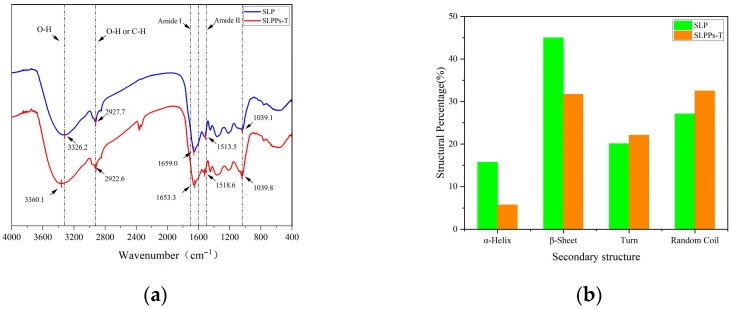
Secondary structure analysis of SLP and SLPPs-T. (**a**) Fourier transform infrared spectra of SLP and SLPPs-T; (**b**) secondary structure content of SLP and SLPPs-T (Note: Values derived from peptide mixtures reflect average conformational populations and should be considered qualitative comparisons).

FTIR spectroscopy provides information on the vibrational states of protein chemical bonds, serving to characterise changes in protein secondary structure and functional groups. As shown in Figure 3a, characteristic absorption peaks appear in the FTIR spectrum between 1700 and 1600 cm^−1^ and between 1600 and 1500 cm^−1^, corresponding to the C=O stretching vibration of the amide I band and the N–H bending vibration of the amide II band. Compared to SLP at 1659.0 cm^−1^ and 1513.5 cm^−1^, SLPPs-T exhibits a red shift in the amide I band absorption peak towards longer wavelengths and a blue shift in the amide II band absorption peak towards shorter wavelengths, indicating that enzymatic hydrolysis induces changes in secondary structure [27]. SLP exhibits a broad peak at approximately 3326.2 cm^−1^, while SLPPs-T shows a blue shift of 33.9 cm^−1^ towards shorter wavelengths, indicating that enzymatic hydrolysis enhances hydrogen bonding. SLP exhibits a secondary peak at 2922.7 cm^−1^, with the SLPPs-T absorption peak shifting to longer wavelengths (red shift), indicating that enzymatic hydrolysis affects C–H symmetric and asymmetric stretching vibrations, suggesting a change in hydrophobicity [28].

As shown in Figure 3b, the secondary structures of SLP and SLPPs-T in aqueous solution were analysed and characterised using circular dichroism (CD) spectroscopy. It should be noted that these spectra represent the average values of complex peptide mixtures, rather than the characteristics of a single purified protein. The data indicate that, following enzymatic hydrolysis, there is a trend towards a reduction in ordered structures (α-helix and β-sheet content), whilst the proportion of random coil conformations increases. Although precise quantitative values are limited due to the heterogeneity of the hydrolysis products, this observed transition is consistent with the unfolding and fragmentation of the native protein structure, which may lead to the exposure of active site domains buried within the protein [29].

#### 3.2.3. Particle Size Distribution

**Figure 4:**
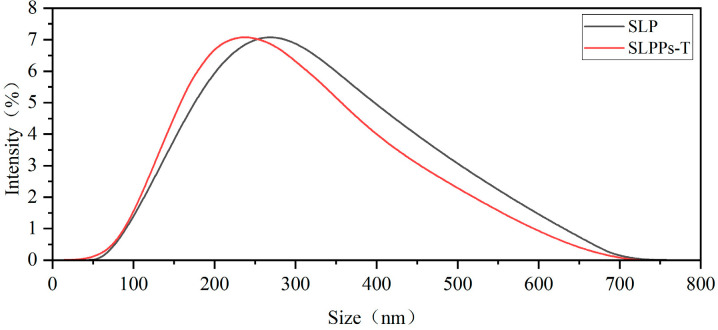
Particle size distribution of SLP and SLPPs-T.

The particle size distribution diagrams for both proteins reveal two distinct peaks at different positions (Figure 4). Instrumental calculations indicate average particle sizes of 261.8 nm for sea buckthorn leaf protein and 237.1 nm for sea buckthorn leaf peptides. Unhydrolysed protein molecules are larger and structurally more intact, hence their concentrated distribution within the 200–300 nm range. Following hydrolysis, macromolecular proteins are cleaved into smaller peptide segments, resulting in a slightly higher proportion in the smaller particle size region (<200 nm) compared to the protein, while the proportion in the larger particle size region (>400 nm) is lower. In the >400 nm range, the proportions of both decrease progressively, but the intensity distribution of peptides declines more rapidly, indicating a marked reduction in their large-particle-size components.

### 3.3. Separation and Purification of Enzymatic Products

Low-molecular-weight peptides are characterised by short amino acid chains, flexible spatial conformation, and the tendency for hydrophobic residues to be exposed. These characteristics enable the peptides to form a stronger affinity with the enzyme active site through hydrogen bonding and hydrophobic interactions [30]. Furthermore, low-molecular-weight fragments possess a larger specific surface area, allowing binding to more enzyme sites per unit mass and thereby enhancing inhibition efficiency [31]. The enzymatic products constitute a mixture of macromolecular proteins and small-molecule peptides. SLPPs-T was separated and purified using Sephadex G-50, yielding a molecular weight–elution volume regression curve of y = −70.511x + 320.6.

**Figure 5:**
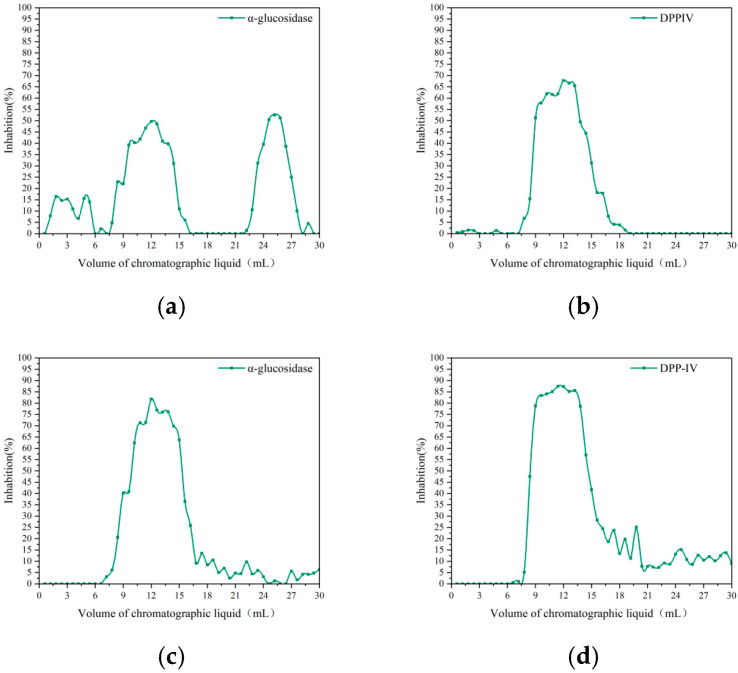
Relationship between inhibitory activity and elution volume of purified eluates from different enzymatic digestion products. (**a**) α-glucosidase inhibitory activity of papain-hydrolysed products; (**b**) DPP-IV inhibitory activity of papain-hydrolysed products; (**c**) α-glucosidase inhibitory activity of trypsin-hydrolysed products; (**d**) DPP-IV inhibitory activity of trypsin-hydrolysed products.

SLPPs-T exhibited a primary elution peak commencing at an elution volume of 10 mL, corresponding to a relative molecular mass range of <25,350 Da. As illustrated in Figure 5, the total elution volume was 30 mL. Using the hyperglycaemic enzyme-inhibitory activity of the eluate as an indicator, it is evident that the activity peaks of purified SLPPs-P and SLPPs-T are primarily concentrated around an elution volume of 12 mL. This indicates that at this elution volume, the products of SLP hydrolysis by both hydrolases exhibit strong inhibitory effects against both hyperglycaemic enzymes, with SLPPs-P demonstrating significantly stronger inhibition than SLPPs-T. Therefore, multiple injections of the SLPPs-P sample were performed, with the active peak fraction collected repeatedly. The purified SLPPs-P was obtained by freeze-drying.

### 3.4. Bioinformatics Characterisation

#### 3.4.1. Sequence Identification Results for Sea Buckthorn Leaf Proteins and Peptides

High-performance liquid chromatography–mass spectrometry (LC/MS-MS) was employed to identify the sequences of SLP and SLPPs-T, yielding 42 protein sequences and 219 peptide sequences, respectively. Table 1 provides information on 10 proteins selected from the identification results. Of the 42 protein entries provided, 21 belong to species of the genus *Hippophae*, whilst the remaining 21 identified proteins do not originate from the *Hippophae* protein database; they are primarily derived from plants of the genus *Elaeagnus*. Consequently, the protein information originally detected in other species within these identification results can be incorporated into the *Hippophae* leaf protein database. Table 2 provides examples of information for 10 peptides. Detailed information on protein and peptide identification is provided in Supplementary Tables S1 and S2, respectively.

#### 3.4.2. Computer-Simulated Peptide Screening

The 219 identified peptides were screened according to the following criteria: 1. The sequence was not modified by functional groups (all 219 peptides met this criterion); 2. The sequence had a bioactivity score higher than 0.8 [32] (21 peptides remained); 3. The sequence was non-toxic and non-allergenic (10 peptides remained); 4. The sequence had not been previously reported (all 10 peptides met this criterion); 5. The peptide chain length was less than 15 (all 10 peptides met this criterion). Following this screening, 10 suitable peptides were identified as candidates for α-glucosidase inhibitors, all of which were 8-mer peptides. Table 3 presents the physicochemical properties of the α-glucosidase candidate peptides.

Furthermore, for DPP-IV inhibitor candidate peptides, the detected peptide segments were analysed using the StackDPPIV tool to predict their DPP-IV inhibitory activity and assign scores. Screening was conducted based on the following criteria: 1. The sequence was not modified by functional groups (all 219 peptides met this criterion); 2. The StackDPPIV score of the sequence was greater than 0.7 (13 peptides met this criterion); 3. The sequence is non-toxic and non-allergenic (seven peptides met this criterion); 4. The sequence has not been previously reported (all seven peptides met this criterion); 5. The peptide chain length is less than 15 (all seven peptides met this criterion). Following this screening, seven suitable peptides were identified as DPP-IV inhibitor candidates. Table 4 presents the physicochemical properties of these DPP-IV inhibitor candidates.

### 3.5. Molecular Docking Analysis

Based on the screened peptide segments, molecular docking was performed with α-glucosidase and DPP-IV respectively. The majority of peptides exhibited spontaneous binding to the proteins. The two segments with the lowest binding energies were selected for solid-phase synthesis, and their IC_50_ values were measured to investigate their hypoglycaemic mechanisms.

**Figure 6:**
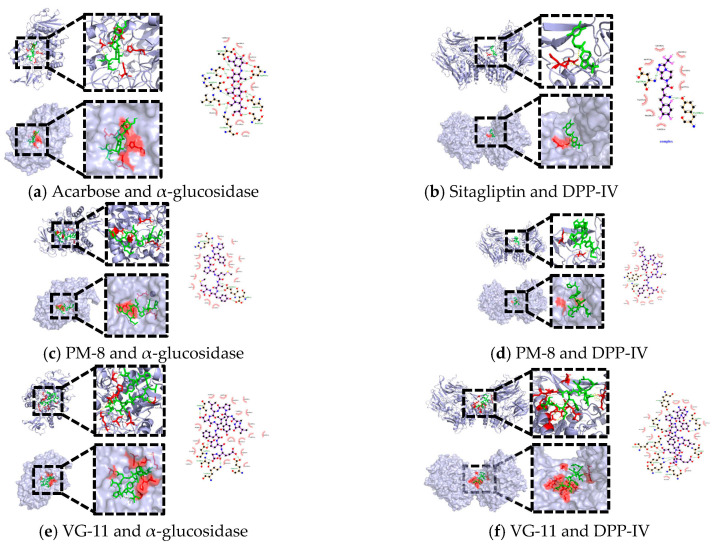
Molecular docking diagram. (**a**) Acarbose with α-glucosidase; (**b**) sitagliptin with DPP-IV; (**c**) PM-8 with α-glucosidase; (**d**) PM-8 with DPP-IV; (**e**) VG-11 with α-glucosidase; (**f**) VG-11 with DPP-IV.

The binding energies of the molecular docking are shown in Table 3 and Table 4, and the docking diagrams are shown in Figure 6. Taking the biological activity of the peptide fragments as an indicator, among the peptide fragments with higher biological activity, PYPPMAYM (abbreviated as PM-8) exhibits a binding energy of −8.6 kcal/mol with α-glucosidase, which is higher than the binding energy of −7.9 kcal/mol between the positive control drug acarbose and α-glucosidase. This suggests that the PM-8 peptide fragment can bind stably to α-glucosidase and may inhibit its activity. Screening based on the StackDPP-IV scores of the peptide segments, among the peptides with higher scores, VAYPLDLFEEG (abbreviated as VG-11) exhibits a docking binding energy of −9.2 kcal/mol with DPP-IV, which is higher than the binding energy of −8.0 kcal/mol for the positive control drug sitagliptin with DPP-IV. This suggests that the peptide segment VG-11 can also bind stably to DPP-IV and has significant potential to inhibit DPP-IV activity. To investigate the comprehensiveness of their activity, the two peptide segments were docked with α-glucosidase and DPPIV respectively; the results showed that both peptide segments bind well to the two receptors.

The results of the molecular docking calculations (Table 5) show that these two new peptides can dock within the internal cavity of α-glucosidase in the computational model, and are predicted to form hydrogen bonds with various amino acid residues within the cavity. A comparison of the computational docking results for acarbose and the two peptide fragments revealed that ARG-315 is a common residue involved in hydrogen bonding with α-glucosidase in all three. Furthermore, all amino acid residues involved in hydrophobic interactions in the acarbose docking model were present in the docking results for PM-8; in the docking results for VG-11, all residues were present except for Ser240 and Pro312. Among residues hydrocarbon-bonded to the peptide segment, ASP-307, ARG-315, ASP-242, ARG-315, PRO-312, SER240, and ASP242 were cited in Han Lu [7]’s literature review, suggesting their potential significance. Wang et al. [33] reported that hydrophobic interactions effectively influence α-glucosidase activity. Furthermore, the number of hydrophobic residues involved in α-glucosidase interactions exceeds that of hydrophilic residues, suggesting that the binding site between the peptide and the enzyme is more likely to be located within an internal hydrophobic region rather than an external hydrophilic region. Based on the predictive results of the aforementioned molecular docking calculations (including the types of interactions and the residues involved), it is speculated that VG-11 may form a more favourable interaction pattern with α-glucosidase. However, this computationally based inference does not imply that VG-11’s actual enzyme-inhibitory activity is necessarily superior to that of PM-8; the true inhibitory effects of both compounds on α-glucosidase still require direct verification through in vitro enzyme activity inhibition assays.

Comparing the relevant amino acid residues in the drug–peptide docking with DPP-IV, PM-8 shares the same residue ALA707 that forms a hydrogen bond with DPP-IV as sitagliptin, while VG-11 shares the same residue ASP-737 that forms a hydrogen bond with DPP-IV as sitagliptin. Furthermore, the amino acid residues exhibiting hydrophobic interactions between sitagliptin and DPP-IV in docking results were present in PM-8’s docking outcome, except for Ala707, and in VG-11’s docking outcome, except for Gln123. The active site of DPP-IV comprises a hydrophobic S1 pocket (Tyr 631, Val 656, Trp 659, Tyr 662, 666, and Val 711) and a charged S2 pocket (Arg 125, Glu 205, 206, Ser 209, Phe 357, and Arg 358) [34]. The catalytic domain spans residues Gln-509 to Pro-766, with Ser-630, His-740, and Asp-708 being crucial catalytic residues [35]. The hydrophobic interactions between PM-8 and VG-11 with DPP-IV show little difference, but their hydrogen bonding patterns diverge significantly. PM-8 forms only two hydrogen bonds with DPP-IV, whereas VG-11 establishes fourteen hydrogen bonds. Notably, six associated amino acid residues within these bonds are located within DPP-IV’s catalytic domain: GLN-718, THR-736, LYS-721, ASP-737, ASP-725, GLU-738. These residues are in close proximity to the catalytic core His-740 and Asp-708. Computational modelling suggests that VG-11 may bind to residues in the catalytic domain, which may be related to its potential mechanism of action. This action may extend to the effects of the hormone insulin and the promotion of insulin secretion, thereby improving glycaemic control [14]. The number of hydrogen bonds predicted by the above calculations (two for PM-8 and 14 for VG-11), together with the interactions between VG-11 and residues in the catalytic domain (such as GLN-718), suggests that, based on theoretical models, VG-11 may have a stronger binding potential for DPP-IV. However, this difference, which is based on computer simulations, does not directly equate to a disparity in their biological activity. The actual inhibitory capacity of VG-11 and PM-8 against DPP-IV must be confirmed through subsequent enzymatic experiments (IC_50_ determination).

It should be noted that all of the above analyses of intermolecular interactions are derived from in silico docking simulations, and the results provide only theoretical predictions of binding modes and affinities. Whilst these computational findings may help to guide subsequent experiments, they cannot serve as direct evidence that the peptide fragments possess enzyme-inhibitory activity or hypoglycaemic properties. Definitive conclusions regarding biological functions, such as enzyme inhibition, must be validated through rigorous in vitro and in vivo experiments.

### 3.6. In Vitro Activity Validation of Peptides

**Figure 7:**
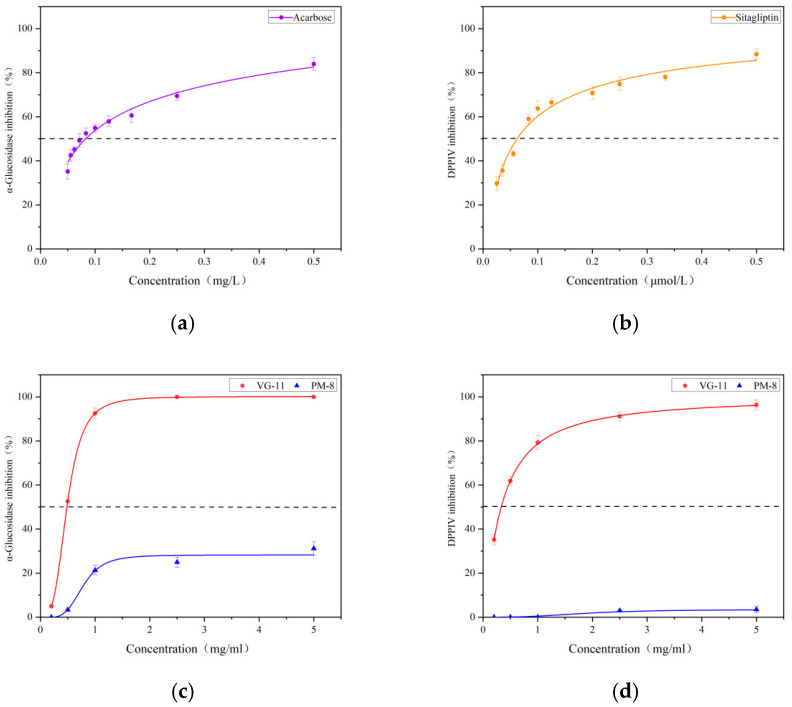
In vitro activity testing of synthetic peptides. (**a**) Acarbose; (**b**) sitagliptin; (**c**) IC_50_ values of PM-8 and VG-11 against α-glucosidase; (**d**) IC_50_ values of PM-8 and VG-11 against DPP-IV.

To validate the inhibitory capacity of the identified peptides against α-glucosidase and DPP-IV, peptides PM-8 and VG-11 were synthesised using solid-phase synthesis. Concurrently, the extracted SLP and SLPPs-T were also evaluated for their inhibitory capacity in vitro, with acarbose and sitagliptin serving as positive controls for α-glucosidase and DPP-IV inhibition, respectively. All IC_50_ data are presented in Table 6, whilst Figure 7 displays the in vitro activity curves for the synthesised peptides. SLP and SLPPs-T exhibited favourable IC_50_ values. Specifically, for α-glucosidase inhibition, SLP demonstrated an IC_50_ of 0.2168 ± 0.023 mg/mL, while SLPPs-T exhibited an IC_50_ of 0.1361 ± 0.017 mg/mL. These values are comparable to those reported by Li [28] et al. for hydrolysed sea buckthorn seed protein. The IC_50_ for the positive control drug acarbose was (0.8331 ± 0.115) × 10^−4^ mg/mL. Regarding DPP-IV inhibitory capacity, SLP exhibited an IC_50_ value of 0.1708 ± 0.015 mg/mL, while SLPPs-T demonstrated an IC_50_ value of 0.1286 ± 0.012 mg/mL. The positive control drug sitagliptin yielded an IC_50_ value of (2.633 ± 0.586) × 10^−5^ mg/mL. The SLPPs-T derived from enzymatic cleavage of SLP exhibited markedly enhanced inhibitory capacity, though it still lagged considerably behind the positive control drug.

Regarding α-glucosidase inhibition by synthetic peptides, PM-8 exhibited only 31.58% inhibition at the maximum tested concentration of 5 mg/mL, whilst VG-11’s IC_50_ value was 0.4865 mg/mL, equivalent to approximately 0.3885 ± 0.015 mM. Regarding DPP-IV inhibitory capacity, PM-8 exhibited an inhibition rate of merely 2.98% at the maximum tested concentration of 5 mg/mL. In contrast, VG-11 demonstrated an IC_50_ value of 0.3270 mg/mL, equivalent to approximately 0.2611 mM. PM-8 exhibited weaker α-glucosidase inhibitory activity than VG-11, while the disparity in DPP-IV inhibition capacity between the two compounds was markedly pronounced. Comparing the homologous literature data on *Hippophae rhamnoides*, Shan, Q [36] et al. isolated and identified the hypoglycaemic peptide FHF from *Hippophae rhamnoides* seed powder, exhibiting an α-glucosidase inhibitory IC_50_ of 3.98 ± 0.16 mM, while IYF demonstrated a DPP-IV inhibitory IC_50_ of 5.32 ± 0.15 mM. VG-11 exhibits superior inhibitory effects on both enzymes compared to FHF and IYF.

It is worth noting that, although the molecular docking results (Table 5) successfully predicted that both PM-8 and VG-11 can bind to the active sites of α-glucosidase and DPP-IV, there are limitations in the accuracy of predicting the differences in their activity. The computational model indicates that the binding energies of VG-11 with α-glucosidase (−10.6 kcal/mol) and DPP-IV (−9.2 kcal/mol) are both significantly lower than those of PM-8 (−8.8 and −8.6 kcal/mol, respectively), and that VG-11 forms a greater number of hydrogen bond interactions. In vitro experimental results partially corroborate this trend: the inhibitory activity of VG-11 against DPP-IV (IC_50_ = 0.2611 mM) far exceeds that of PM-8 (which exhibits an inhibition rate of only 2.98% at 5 mg/mL), consistent with the prediction that VG-11 forms as many as 14 hydrogen bonds with the catalytic domain of DPP-IV in this docking model.

However, this computational model failed to effectively explain the difference in their inhibitory effects on α-glucosidase. Although VG-11 exhibited a binding energy far superior to that of PM-8 in the docking analysis, in vitro experiments showed that VG-11 (IC_50_ = 0.3885 mM) demonstrated only limited inhibitory activity, whilst PM-8 exhibited virtually no inhibitory effect at the same concentration. This indicates that molecular docking scoring functions based on static crystal structures have inherent limitations in predicting the dynamic interactions between flexible peptides and proteins; in particular, minor differences in binding energy (approximately 1.8 kcal/mol in this study) cannot be directly equated with proportional changes in in vitro inhibitory activity. This discrepancy between computational and experimental results highlights the challenges faced by current in silico screening methods in accurately predicting the structure–activity relationship of peptide segments. Future research should incorporate methods such as molecular dynamics simulations to more accurately model solvent effects and the dynamic conformational changes in protein-ligand complexes, thereby improving the accuracy of activity predictions.

## 4. Conclusions

This study confirms that peptides with high α-glucosidase and DPP-IV inhibitory activity can be prepared from sea buckthorn leaf protein (SLP) via enzymatic hydrolysis, with SLPPs-T, obtained by trypsin hydrolysis, exhibiting significant inhibitory effects. Through a comprehensive analysis involving separation and purification, peptide identification, and computer-aided virtual screening, a novel hypoglycaemic peptide, VG-11, was successfully identified. Solid-phase synthesis and in vitro inhibition assays confirmed that the half-maximal inhibitory concentrations (IC_50_) of VG-11 against α-glucosidase and DPP-IV were 0.3885 ± 0.015 mM and 0.2611 ± 0.021 mM, respectively. However, a comparison of computational and experimental data for PM-8 and VG-11 revealed that there is no simple linear relationship between docking binding energy and in vitro IC_50_ values; differences in binding energy do not equate to equivalent differences in in vitro activity. This finding suggests that when using molecular docking for the screening of bioactive peptides, binding energy should not be used as the sole criterion; instead, the interactions of key residues within the peptide–enzyme complex should be comprehensively considered, with final validation relying on in vitro experiments. The above results provide a theoretical basis for the efficient screening of hypoglycaemic peptides using computational methods. Furthermore, the high inhibitory activity of SLPPs-T indicates that SLP holds significant potential in the development and application of natural hypoglycaemic peptides, helping to reduce waste of sea buckthorn leaf protein resources and enhance the comprehensive utilisation value of sea buckthorn.

## Figures and Tables

**Table 1:** Partial results of SLP identification.

ID	Protein Name	Number of Amino Acids	Source Species	Molecular Weight
1	ribulose-1,5-bisphosphate carboxylase/oxygenase large subunit	475	*Hippophae rhamnoides* subsp. *mongolica*	52.71 kDa
2	ATP synthase CF1 alpha subunit	507	*Hippophae rhamnoides* subsp. *caucasica*	55.39 kDa
3	ATP synthase CF1 beta subunit	498	*Hippophae rhamnoides* subsp. *caucasica*	53.66 kDa
4	zeta-carotene desaturase, partial	151	*Elaeagnus umbellata*	16.91 kDa
5	RNA polymerase beta subunit	1379	*Elaeagnus loureirii*	156.82 kDa
6	RNA polymerase beta″ subunit	1381	*Elaeagnus bambusetorum*	157.07 kDa
7	ribosomal protein L2	274	*Elaeagnus moorcroftii*	29.84 kDa
8	hypothetical chloroplast RF19	1858	*Elaeagnus angustifolia*	222.44 kDa
9	ATP synthase subunit 1	509	*Hippophae gyantsensis*	55.28 kDa
10	ATP synthase CF1 epsilon subunit	133	*Elaeagnus moorcroftii*	14.52 kDa

**Table 2:** Partial Results of SLPPs-T peptide segment identification.

ID	Sequence	Number of Amino Acids	Mass	Source Species
1	TQDWVSLPGVLPVA	14	1480.7926	*Elaeagnus macrophylla × Elaeagnus pungens*
2	TAIDIGILR	9	970.58113	*Elaeagnus moorcroftii*
3	DLFEEGSVTN	10	1109.4877	*Elaeagnus macrophylla × Elaeagnus pungens*
4	VAYPLDLFEEG	11	1251.6023	*Elaeagnus macrophylla × Elaeagnus pungens*
5	LSLYTPAG	8	820.43307	*Hippophae salicifolia*
6	FLGLFWMY	8	1075.5201	*Elaeagnus moorcroftii*
7	FPKNWLTD	8	1019.5076	*Elaeagnus macrophylla*
8	IMNNVKVY	8	979.51609	*Elaeagnus macrophylla*
9	ITNELQLY	8	992.51786	*Elaeagnus moorcroftii*
10	HISYMKKR	8	1061.5804	*Hippophae rhamnoides* subsp. *caucasica*

**Table 3:** Candidate peptides for α-glucosidase inhibition.

Bioactivescore	Sequence/ Positive Drugs	Hydrophobicity	Hydrophilicity	Molar Mass (g/mol)	α-Glucosidase Docking Binding Energy (kcal/mol)
0.92	PYPPMAYM	−0.22	−0.96	969.28	−8.6
0.93	RFAFMLYC	0.07	−1.11	1050.4	−8.5
0.97	FLGLFWMY	0.39	−1.95	1076.44	−8.2
0.95	WAFFLARI	0.2	−1.25	1023.35	−7.9
0.98	AWMFLFGH	0.3	−1.56	1008.32	−7
0.89	QPFMRWRD	−0.47	0.25	1135.41	−6.9
0.94	SIANYMWF	0.17	−1.41	1031.3	−6.7
0.93	SRNMLFFR	−0.3	−0.2	1070.38	−6.3
0.98	AFSLMFLF	0.39	−1.58	975.32	−4.6
0.81	IGPLLPRG	0.03	−0.3	822.14	−1.9
—	Acarbose	—	—	—	−7.9

Note: The last column of the table shows the binding energy of the positive drug acarbose docking with α-glucosidase.

**Table 4:** DPP-IV screening inhibitor candidate peptides.

StackDPPIV Score	Sequence/ Positive Drugs	Hydrophobicity	Hydrophilicity	Molar Mass (g/mol)	DPP-IV Docking Binding Energy (kcal/mol)
0.87	PYPPMAYM	−0.22	−0.96	969.28	−8.6
0.77	SSTGTWTT	−0.71	−0.55	839.96	−8.3
0.75	GVIAAGDA	1.23	−0.22	672.84	−8
0.72	LNYYTPEY	−1.18	−0.74	1062.25	−7.6
0.72	VAYPLDLFEEG	0.24	−0.13	1252.54	−9.2
0.72	YQIEPVAG	0.02	−0.36	876.09	−8.1
0.7	VALDSGVP	0.96	−0.25	756.96	−7.9
—	Sitagliptin	—	—	—	−8.0

Note: The last column of the table shows the binding energy of the positive drug Sitagliptin docking with DPP-IV.

**Table 5:** Molecular docking binding data for positive drugs, synthetic peptides, and receptors α-glucosidase, DPP-IV (displaying binding energy within the enzyme active site, hydrogen bond interactions, hydrophobic interactions).

Ligand	Receptor (PDB ID)	No. of H-Bonds	Binding Energy (kcal/mol)	Shortest H-Bond Length (Å)	Hydrogen Bond Interactions (Distance Å)	Hydrophobic Interactions	Salt Bridges	π-Stacking
Acarbose	3AJ7	4	−7.9	2.4	Asp-307, Arg-315, His-280, Asp-242	Val308, Ser304, Pro312, His280, Ser240, Tyr158, Phe314	His280	None
Sitagliptin	5J3J	2	−8	2.7	Ala-707, Asp-737	Glu738, Ala707, Trp124, Phe240, Val252 Asp737, Lys122, Gln123	None	None
PYPPMAYM	3AJ7	7	−8.8	1.9	Glu-332, Gln-279, Ser-304, Arg-315	Asp242, Pro312, Asp307, Val308, Asp325, Ala329, Ile328, Ser304, Ala281, Phe303, His280, Gln279, Tyr158, Ser240, Phe314	None	Phe303
PYPPMAYM	5J3J	2	−8.6	2.5	Ala-707, Ser-242	Lys250, Val252, Trp124, Phe240, Gln123, Asp709, Asp739, Asp737, Glu738, Ser744, Lys122, Val121	None	Phe240
VAYPLDLFEEG	3AJ7	9	−10.6	2	Thr-310, Pro-312, Leu-313, Lys-516, Ser-240, Asp-242, Ala-281	His280, Leu313, Tyr158, Val232, Phe314, Tyr316, Arg315, Asp307, Pro312, Val319, Thr310, Gly309, Val308, Ser304, Glu332, Ile328, Ala329, Ser282	Lys156	None
VAYPLDLFEEG	5J3J	14	−9.2	2.1	Lys-250, Asp-243, Ala-244, Gln-718, Thr-736, Lys-721, Asp-737, Asp-725, Glu-738, Gln-123, Lys-122	Ala707, Asp739, Ser744, Glu738, Asp737, Ala722, Met689, Phe240, Val121, Trp124, Val252, Gln123	Lys721	Phe240

**Table 6:** Half-inhibitory concentrations of peptides and positive control drugs against α-glucosidase and DPP-IV.

Sequence/Name	α-Glucosidase Inhibition/IC_50_	DPP-IV Inhibition/IC_50_
SLP	0.2168 ± 0.023 mg/mL	0.1708 ± 0.015 mg/mL
SLPPs-T	0.1361 ± 0.017 mg/mL	0.1286 ± 0.012 mg/mL
PM-8	31.58% (5 mg/mL)	2.98 % (5 mg/mL)
VG-11	0.4865 ± 0.019 mg/mL (0.3885 ± 0.015 mM)	0.3270 ± 0.026 mg/mL (0.2611 ± 0.021 mM)
Acarbose	(0.8331 ± 0.115) × 10^−4^ mg/mL (1.2901 ± 0.178) × 10^−4^ mM	-
Sitagliptin	-	(2.633 ± 0.586) × 10^−5^ mg/mL (6.529 ± 1.453) × 10^−5^ mM

## Data Availability

The original contributions presented in this study are included in the article/Supplementary Materials. Further inquiries can be directed to the corresponding authors.

## Supplementary Materials

The following supporting information can be downloaded at: https://www.mdpi.com/article/10.3390/foods15091489/s1.
