# Supplementary material for: A Novel Peptide Derived from Sea Buckthorn Leaves: Enzymatic Preparation, Dual Inhibitory Activity Against α-Glucosidase and DPP-IV, and Its Molecular Mechanism
Source: Foods. 2026 Apr 24;15(9):1489. doi: 10.3390/foods15091489 (PMC13163793; doi:10.3390/foods15091489)
Supplement: Supplementary file 1 [file foods-15-01489-s001.zip › List description.pdf]

Table S1. List of protein identifications

Header	Definition	Description
Protein ID	Proteome accession number	The identification numbers of each protein within a proteome (corresponding to protein IDs in the database). A proteome refers to a group of proteins that share such a high degree of sequence conservation or homology that they cannot be further distinguished by unique peptides identified via mass spectrometry; these proteins are grouped together as a proteome. Proteins within a proteome may also represent duplicate entries for the same protein in the database.
Fasta header information	Protein description	Functional descriptions of proteins in the database based on protein sequences.
Number of proteins	Number of proteins	The number of proteins contained within the proteome
Peptides	Number of peptides	The number of peptides used for qualitative analysis
Unique peptides	Number of unique peptides	The number of unique peptides used for quantitative analysis. Definition of unique peptides: peptide sequences specific to a particular proteome, capable of distinguishing it from other proteomes, are referred to as the unique peptides of that proteome. The greater the number of unique peptides, the higher the accuracy of the quantification.
Sequence coverage [%]	Peptide coverage	The proportion of identified amino acids relative to the total number of amino acids in the protein. A sequence coverage value greater than zero indicates that the protein has been detected in the sample, whilst a value of zero indicates that it has not been detected in the sample.
Molecular weight [kDa]	Molecular weight	The theoretical molecular weight of the protein.
Sequence length	Sequence length	The sequence length of the protein.
LFQ intensity	LFQ peak intensity	Protein signal intensity corrected by the LFQ algorithm. Used as a relative quantification result for the protein, typically for screening differentially expressed proteins between samples.

Table S2. List of protein identifications

Header	Definition	Description
Sequence	Peptide sequence	Sequence of the identified peptide
Mass	Peptide molecular weight	Theoretical molecular weight of the peptide
Leading razor protein	Identifier of the top-ranked protein in the protein group to which the peptide corresponds	The ID of the top-ranked protein in the protein group to which the peptide corresponds. A protein group refers to a set of proteins that share such a high degree of sequence conservation or homology that peptides identified by mass spectrometry (unique peptides) cannot distinguish them further; these proteins are grouped together. Proteins within a single protein group may also represent duplicate entries for the same protein in the database.
Unique (Groups)	Whether it is the only peptide in that protein group	Whether the peptide is unique to this protein group. Definition of unique peptides: Peptide sequences that are specific to a particular protein group and can distinguish it from other protein groups are referred to as the unique peptides of that protein group. The greater the number of unique peptides, the higher the accuracy of quantification.
Charges	Charge	The charge of the peptide.
Experiment	Number of times the peptide was detected in the sample	The number of times the peptide has been detected in the sample. A value greater than zero indicates that the peptide has been detected in the sample, whilst a value of zero indicates that it has not been detected in the sample.
Intensity	Peak intensity	The signal intensity of the peptide. This serves as the relative quantification result for the peptide and is typically used to screen for differentially expressed peptides between samples.
